# Changes in Blood Profile from Steady State in Patients with Sickle Cell Anemia Admitted for Vaso-occlusive Crisis and Acute Chest Syndrome

**DOI:** 10.1155/2020/3656717

**Published:** 2020-08-25

**Authors:** Timothy Klouda, Deepti Raybagkar, Bruce Bernstein, Nataly Apollonsky

**Affiliations:** ^1^Pediatrics, St. Christopher's Hospital for Children, Philadelphia, PA, USA; ^2^Hematology, St. Christopher's Hospital for Children, Philadelphia, PA, USA

## Abstract

Close to half of all patients with sickle cell disease (SCD) will have at least one episode of acute chest syndrome (ACS) during their lifetime. Multiple cells and molecules involved with the inflammatory cascade play a role in the development of ACS. We found that patients with SCD who developed ACS as a complication of a vaso-occlusive crisis (VOC) had a significant increase in leukocytes and decrease in platelets from their steady state when compared with a separate admission for VOC without ACS development. No significant change from steady state hemoglobin or reticulocyte count was noted between the two admissions. These results indicate that trending laboratory markers may be useful to predict patients at risk for ACS development.

## 1. Introduction

ACS is the most common pulmonary complication in patients with sickle cell anemia [1, 2]. More than half of all patients with homozygous HbS experience at least one episode of ACS in the first decade of life [1, 2]. It is commonly seen as a complication in hospitalized patients admitted for VOC and is the leading cause of death among patients with SCD [1–3].

The mechanism of VOC leading to ACS development is thought to be multifactorial. Infection is the most common cause of ACS in pediatric patients, with a pathogen such as *Chlamydia pneumonia, Mycoplasma pneumonia*, and respiratory syncytial virus (RSV) identified in approximately 35% of ACS cases. Lower respiratory tract infections can result in hypoxia and inflammation, predisposing patients to hemolysis and ACS development [2]. Dehydration and stress cause inflammation and polymerization of hemoglobin S, resulting in hemolysis and enhanced leukocyte interactions within vascular endothelium. In response, there is a release of inflammatory cytokines within the airway vasculature. Inflammation upregulates the expression of adhesion proteins, which results in binding of red blood cells, leukocytes, and platelets to the endothelium [4, 5]. Increased adhesion of molecules to the endothelium leads to microvascular occlusion and ultimately ACS development [1–3]. Fat emboli from infarcted and necrosed bone marrow during a crisis can also travel to the lungs, causing infarction and development of ACS [1]. Additionally, during VOC, patients are often in severe pain, resulting in shallow breathing with minimal chest wall expansion, and together with high doses of opioids, it could lead to hypoventilation, with the formation of atelectasis, and increase risk for the development of ACS [6].

At a steady state, patients with SCD have increased leukocyte and platelet activity predisposing them to inflammation and SCD related complications [7, 8]. An increase in acute phase reactants and inflammatory markers can be detected during crises and predict severity [9, 10]. Changes from steady state in frequently monitored laboratory markers during hospitalizations could help further describe the pathophysiology behind ACS development and potentially identify patients admitted with a VOC at risk for ACS development, resulting in decreased hospital costs and length of stay and improving long-term outcome.

We hypothesized that patients with SCD admitted with a VOC complicated by ACS will have significant changes in laboratory data from steady state compared with VOC alone. We predict laboratory changes will reflect ongoing inflammation, including a decrease in platelet count and hemoglobin, with an increase in white blood cell count. The secondary aim of this study was to determine risk factors for recurrent episodes of ACS.

## 2. Methods

We conducted a retrospective, cohort chart review of all patients with SCD hospitalized with VOC to a single, urban, pediatric hospital over a 6-year period. This study was approved by the Drexel University Institutional Review Board.

Patients with an ICD code for SCD and VOC admitted between January 1, 2012, and February 1, 2018, who had a separate admission for both VOC and VOC complicated by ACS were included in the study. Laboratory data from the subject's separate hospital admissions were organized into two groups for comparison. One group contained the subject's changes in laboratory data from steady state during the VOC admission and the other group contained the subject's changes in laboratory data from steady state during the VOC admission complicated by ACS. Subjects who did not have any laboratory tests performed on admission were excluded from the study. The first set of laboratory data obtained in the emergency department (ED) was used for analysis, with timing and specific labs obtained up to the discretion of the ED physician and admitting hematologist. ACS at our institute is defined as a new pulmonary infiltrate on chest X-ray accompanied by two out of three of the following findings: presence of fever (>38.5°C), chest pain, and respiratory symptoms (hypoxemia, tachypnea, increased work of breathing, etc.) [1]. Potential subjects presentation and hospital course were reviewed for inclusion and confirmation of ACS diagnosis based on attending hematologist's progress. ACS occurring any time during the admission was included in the analysis. Any patient with symptoms confirmed by an alternate diagnosis was excluded, such as a pulmonary embolism (PE). All patients with positive blood cultures were excluded due to possible cofounding of results.

Steady state data was obtained during a routine hematology clinic visit within 12 months of the admission. Subjects who had missing data were excluded from that specific analysis. Data collected for analysis included white blood cell (WBC) count, hemoglobin (Hgb), absolute reticulocyte count (ARC), platelet count, absolute neutrophil count (ANC), absolute eosinophil count (AEC), and oxygen saturation.

Recurrent ACS was defined as two or more lifetime episodes of ACS. Clinical data collected for potential risk factors for recurrent ACS included history of asthma, splenectomy, past admission for VOC, and abnormal polysomnography. An abnormal polysomnography was defined as an abnormal interpretation from a pulmonologist. This included but was not limited to nocturnal hypoxemia and any degree of obstructive or central sleep apnea. Asthma was determined if patients carried the diagnosis or ICD code in their EMR.

Statistical analysis was performed using the Statistical package for the (SPSS) software (version 24.0). Paired *t*-tests were used to explore differences in laboratory changes from baseline (steady state) to VOC-ACS admissions versus VOC–no ACS admissions. The issue of multiple testing in the paired *t*-tests was addressed by calculating the false discovery rate via the Benjamini–Hochberg procedure with the maximum discovery limit set at 10% [11–13]. This procedure is appropriate for exploratory studies in that it relaxes criteria for type 1 errors in order to increase the number of alternative hypotheses which have small *p* values but are rejected and therefore lost to the literature or as candidates for further study. Chi-square analysis was used to evaluate risk factors for ACS between those with recurrent ACS, defined by two or more lifetime ACS episodes, and those with a single episode or none. Critical significance level was defined as *p* < 0.05, as adjusted with the Bonferroni correction.

## 3. Results

Forty-five subjects were included in the analysis. The median age was 12.4 years, ranging from 2 to 26 years; 28 (62.2%) of the subjects were male; 37 subjects (82.2%) were SS genotype, with five (11.1%) patients with SC genotype, two (4.4%) patients with Sb^+^ genotype, and one patient (2.2%) with Sb^0^ genotype. Twenty-two subjects (48.8%) had a prescription for hydroxyurea on admission. The average BMI-for-age percentile of the 45 subjects was 45.1%.

When comparing the VOC complicated by ACS group with the uncomplicated VOC, the Benjamini–Hochberg false discovery rate (B-H FDR) identified a significantly greater increase in WBC (6.8 K cells/*μ*L vs. 4.1 K cells/*μ*L; *p*=0.025. B-H FDR *p*=0.06) and ANC (6.0 K cells/*μ*L vs. 3.7 K cells/*μ*L; *p*=0.043, ANC B-H FDR *p*=0.07) when compared with the VOC group. Steady state oxygen saturations were found to be similar between the ACS and VOC groups (98.2% vs. 98.4%). However, a significant decline was noted in VOC complicated by ACS compared with VOC alone (−2.2% vs. −0.26%; *p*=0.011, B-H *p*=0.06). No statistically significant changes in Hgb, AEC, or ARC were found. Data extracted from the ACS and VOC admission along with the comparison to steady state can be seen in Table 1.

When comparing risk factors for recurrent ACS, subjects with two or more lifetime ACS episodes were more likely to have >3 VOC in the past 12 months (38.9% vs. 3.8%; *p*=0.003) and an abnormal polysomnography (50.0% vs. 22.2%; *p*=0.052) compared with those who had one or no episodes of ACS. A history of splenectomy or asthma was not found to be a risk factor for recurrent ACS. Results can be seen in Table 2.

## 4. Discussion

To our knowledge, this is the first study comparing the change in laboratory data from steady state in patients when they are admitted with VOC to when they are admitted with a VOC complicated by ACS. Our findings suggest that patients with SCD who develop ACS as a complication of VOC have a greater decrease in platelets and increase in WBC count from steady state on admission compared to when they are admitted for an uncomplicated VOC.

Platelets can act as an acute phase reactant, having increased adhesive properties [13]. This proinflammatory state predisposes patients to a crisis. Inflammation results in endothelial activation, causing platelet aggregation and consumption [13, 14]. The cause of consumption is unknown and may be due to the inflammation itself, infection, viral suppression, and splenic destruction or from a DIC-like picture. While the exact role of platelets during VOC and ACS is not fully understood, platelet thrombi have been detected during autopsy in adult patients with ACS. A platelet count <200 × 10^9^/L has been found to be an independent predictor of respiratory failure and neurologic complications in patients with ACS. A rapid decline in platelet count also commonly precedes a rapidly progressive course [8, 15]. However, no studies until now have compared the degree of platelet consumption and the progression of VOC to ACS. Our data suggests the consumption of platelets during inflammation may have a significant role in ACS development.

Neutrophil activation and adhesion have been well documented in literature playing a significant role in the development of VOC [16, 17]. Neutrophils and other leukocytes expressing E-selectin ligand bind to E-selectin on endothelium, resulting in microvascular obstruction and a positive feedback loop [5]. This increase in circulating leukocytes and neutrophils may lead to the progression of VOC to ACS, and trending values during admission along with other acute phase reactants may predict ACS development. Changes in leukocytes and neutrophils are affected by multiple stimuli, such as medications, infections, and inflammation. While our results show an increase in leukocytes and WBC count from baseline in patients who developed ACS while experiencing VOC, this change potentially could be from a secondary process such as a viral infection. Regardless, the increase in leukocyte count from steady state during admission for VOC complicated by ACS compared with uncomplicated VOC suggests an ongoing, progressive inflammatory response. Further studies and research are needed before this finding can be used clinically.

Due to SCD primarily being a disorder of red blood cell destruction, hemolysis is largely considered to be a significant factor in the progression of SCD complications. Stress leading to the sickling of red blood cells causes increased aggregation, viscosity, and microvascular obstruction that can result in pain and ACS development [1–3]. Hemolysis can also lead to the release of heme and other molecules that can trigger an inflammatory cascade, leading to the development of complications [16, 18]. While inflammation and hemolysis can drive one another during an acute crisis, our results showing no difference in hemoglobin and reticulocyte count between the two groups hypothesize that the inflammatory cascade is exacerbated by more than just byproducts of hemolysis. Further studies looking at inflammation itself at baseline and during acute complications however are needed before a distinction between the two processes can be confirmed.

Hypoxemia is a common finding in ACS development [1, 2]. Hypoventilation secondary to pain can result in an increased respiratory rate and decreased tidal volume [1]. This leads to a perfusion ventilation (V/Q) mismatch, predisposing patients to atelectasis and the risk of ACS development. High doses of morphine given during a VOC can decrease respiratory drive and predispose to ACS development [19]. Hypoxia inhibits nitric oxide (NO) production from the endothelium, which is an inhibitor of vascular cell adhesion molecule-1 (VCAM-1). VCAM-1 binds to sickle cell erythrocytes causing intrapulmonary adhesion of erythrocytes and other cells. The ratio of soluble VCAM-1 to NO metabolites during ACS is significantly higher compared with steady state or during VOC [5, 7]. Pulse oximetry has been confirmed to be a reliable marker of arterial oxygen saturation in previous studies, suggesting that trends from steady state may be useful to predict molecular changes predisposing patients to ACS [20]. While our data revealed a difference in oxygen saturation between the ACS and VOC group, 18 total subjects did not have steady state oxygen saturations recorded at their outpatient appointment within one year of admission and so were excluded from analysis, decreasing our sample size.

Patients with sleep disorder breathing (SDB), including obstructive sleep apnea (OSA) or nocturnal hypoxemia, are predisposed to chronic hypoxia that can lead to baseline inflammation and SCD complications such as ACS. No increased risk was associated with a diagnosis of asthma or splenectomy, which contradicts what prior studies have found [21]. A possible reason for this difference could be misdiagnosing patients with asthma, as formal pulmonary functions tests were not used for diagnosing asthma in many patients, and wheeze may be due to airway hypersensitivity or inflammation rather than asthma.

This study had several limitations. First, as a retrospective study, laboratory testing and treatment was performed at the discretion of the treating clinician and there was room for human error and misinterpretation. Patient laboratory changes may also be due to a response to another process. Second, the laboratory data collected during hospitalization and steady state is a snapshot in time and may not represent accurate trends, as patients can be within different stages of progression from VOC to ACS when data was collected. Time of laboratory tests in relation to admission or development of ACS was also not analyzed. Third, although all patients receive the same standard of care, compliance with medications and interventions that decrease the incidence of ACS was not analyzed, including the frequency of incentive spirometry, hydroxyurea, and positive pressure ventilation. Opioid doses and sedatives given to patients were also not accounted for and could have predisposed patients to ACS development due to hypoventilation and atelectasis formation. Finally, other laboratory data such as LDH and bilirubin were not routinely collected and thus could not be analyzed due to small sample size, giving an incomplete picture of all laboratory data and trends.

Findings of this study demonstrate that increased platelet consumption and acute on chronic inflammation may contribute to the progression of VOC to ACS. In VOC admission complicated by ACS, studies demonstrated a larger decrease in platelets and increase in WBC count from steady state compared with VOC alone. While platelet and WBC count cannot be used alone to predict development of ACS, future large, prospective and multicenter studies to further understand the pathophysiology of ACS and determine if there are laboratory changes from steady state can be used in a clinical setting.

## Figures and Tables

**Table 1:** Numerical change and analysis from steady state between VOC admission and VOC admission complicated by ACS.

Lab	Sample size	Mean difference in ACS admission and steady state	Mean difference in VOC admission and steady state	Difference between ACS and VOC	95% confidence interval	*p* value B-H *p* value
WBC count (K cells/*μ*L)	43	6.8	4.1	2.71 (±7.62)	[0.36, 5.05]	0.025 0.058^*∗*^
ANC (K cells/*μ*L)	39	6.0	3.7	2.27 (±6.77)	[0.77, 4.47]	0.043 0.058^*∗*^
Platelet count (K cells/*μ*L)	40	−108.0	−44.0	−63.90 (±164.4)	[−116.44, −11.31]	0.019 0.058^*∗*^
AEC (K cells/*μ*L)	37	−0.07	−0.16	0.89 (±0.42)	[−0.05, 0.23]	0.212 0.294
Hgb	41	−0.67	−0.35	−0.32 (±1.75)	[−0.87, 0.23]	0.252 0.294
ARC (K cells/*μ*L)	34	0.06	0.05	0.006 (±0.15)	[−0.05, 0.06]	0.827 0.827
Oxygen saturation (%)	27	−2.20	−0.26	−1.96 (±3.73)	[−3.44, −0.49]	0.011 0.058^*∗*^

^*∗*^Significant by Benjamini–Hochberg false discovery rate.

**Table 2:** Risk factors for two or more lifetime ACS admissions.

	>3 VOC in the past 12 months	Abnormal sleep study	Asthma	Splenectomy
>2 lifetime ACS episodes (*n* = 18)	7 (38.9%)	9 (50.0%)	13 (72.2%)	6 (18.5%)
0-1 lifetime ACS (*n* = 27)	1 (3.8%)	6 (22.2%)	15 (55.6%)	6 (33.3%)
*p* value	0.003^*∗*^	0.053^*∗∗*^	0.208	0.257

^*∗*^Bonferroni corrected *p*=0.009. ^*∗∗*^Bonferroni corrected *p*=0.159.

## Data Availability

Data are available upon request.
